# Editorial: Pharmacoepidemiology in infectious diseases

**DOI:** 10.3389/frabi.2026.1965761

**Published:** 2026-09-03

**Authors:** Cesar Henriquez-Camacho

**Affiliations:** 1 Department of Internal Medicine, Hospital Universitario de Mostoles, Madrid, Spain; 2 Department of Medical Specialties and Public Health, Faculty of Health Sciences, Universidad Rey Juan Carlos, Madrid, Spain; 3 Faculty of Medicine, Universidad Francisco de Vitoria, Madrid, Spain

**Keywords:** epidemiology, infectious diseases, outbreaks, pharmacoepidemiology, pharmacology, public health

## Introduction

This Research Topic includes six original articles that explore different aspects of pharmacoepidemiology, ranging from early warning outbreak surveillance, large-scale pharmacovigilance, therapeutic optimization based on drug resistance mutations, and vaccine effectiveness.

Post-marketing safety evaluations are essential for identifying rare or long-term adverse events (AEs) that are typically excluded from randomized trials. Two articles utilized the FDA Adverse Event Reporting System (FAERS) to characterize the safety profiles of newer antibiotics. Xie et al. conducted a comprehensive pharmacovigilance analysis of oritavancin, a long-acting lipoglycopeptide. While corroborating known signals such as chills and infusion-related reactions, the study identified multiple underemphasized or unlabeled signals, including dyspnea, chest discomfort, and anaphylactic reactions. Their findings underscore the need for intensified vigilance, particularly during the first few days of treatment, when the majority of AEs occur. Li et al. utilized disproportionality and Bayesian analysis to explore coagulation dysfunction associated with novel tetracycline-class drugs: tigecycline, eravacycline, and omadacycline. They identified reporting signals for tigecycline and eravacycline, such as hypofibrinogenemia and prolonged prothrombin time, which emerged as the predominant coagulation abnormalities associated with the three antibiotics. Conversely, omadacycline showed a generally more favorable coagulation profile, although discrete positive signals for hypofibrinogenemia were noted in subgroup analyses.

Li et al. explored the effectiveness of using over-the-counter pharmaceutical sales as a timely indicator for predicting respiratory infectious disease trends in Pinghu, China. This approach allows public health authorities to detect potential outbreaks before confirmed hospital data are available, facilitating better medical resource allocation and more rapid epidemic responses. However, they noted that this information should be integrated with other data sources, such as school absence records and online search queries, to improve early monitoring and overall surveillance.

Zhang et al. investigated drug resistance mutations in people living with HIV experiencing low-level viremia in Chongqing, China. By employing a combined plasma RNA and proviral DNA genotyping strategy, they detected mutations in 26.32% of the cohort, predominantly against non-nucleoside reverse transcriptase inhibitors (NNRTIs). Their study identified that low baseline CD4+ T-cell counts (<100 cells/uL) were significantly associated with resistance, demonstrating that the genotyping strategy enhanced resistance detection, even when a low viral load was present, underscoring its value for resistance surveillance and optimizing clinical management.

Regarding adjunctive management, Nie et al. performed a meta-analysis evaluating the role of adjunctive therapies (nasal irrigation, steroid-eluting implants, and concurrent dental management) for recurrent rhinosinusitis. Their analysis demonstrated that these modalities demonstrated a 30-50% improvement in symptom scores (e.g., SNOT-22) and reduced recurrence rates compared to conventional treatment alone, supporting a multimodal, patient-centered approach to disease management.

Finally, the long-term impact of immunization programs is a vital area of study. Liu et al. analyzed the epidemiological characteristics and vaccine effectiveness (VE) of pertussis vaccination in Linping District, China. While the DTaP vaccine series provided high protection (>90% effectiveness) for children through the preschool age, the study noted a distinct spring peak in incidence. These findings highlight delayed or missed vaccination as a key risk factor for infant cases and suggest that waning immunity may necessitate the evaluation of booster doses at the school age.

## Conclusion

Pharmacoepidemiology provides indispensable insights into the real-world use and impact of infectious disease interventions, thereby ensuring safe, effective, and equitable healthcare strategies. Its integration with emerging technologies and global data networks will further enhance preparedness for future health crises.

